# What care do people with dementia receive at the end of life? Lessons from a retrospective clinical audit of deaths in hospital and other settings

**DOI:** 10.1186/s12877-023-04449-1

**Published:** 2024-01-09

**Authors:** Zoi Triandafilidis, Sally Carr, Daneill Davis, Simon Chiu, Lucy Leigh, Sarah Jeong, Daniel Wong, Jacinta Hensby, Suzanne Lewis, John Attia, Nicholas Goodwin

**Affiliations:** 1 Central Coast Research Institute (CCRI) for Integrated Care, Gosford, NSW Australia; 2 The University of Newcastle, Gosford, NSW Australia; 3 Central Coast Local Health District, Gosford, NSW Australia; 4 Hunter Medical Research Institute, Newcastle, NSW Australia; 5 The University of Sydney, Sydney, NSW Australia

**Keywords:** End of life, Palliative care, Medical records review, Dementia, Quality improvement

## Abstract

**Background:**

The need for better end-of-life care for people with dementia has been acknowledged. Existing literature suggests that people dying with dementia have less access to palliative care, yet little is known about the care provided to people with dementia at the end of life. This study aimed to establish evidence related to end-of-life care for people dying with dementia in hospital compared to other settings.

**Methods:**

A retrospective clinical audit of people who had a diagnosis of dementia and had accessed services within a local health district, who died between 2015 and 2019, was conducted. A total of 705 people were identified, and a subset of 299 people randomly selected for manual audit. Chi-square *p*-values were used to compare the place of death, and a t-test or non-parametric test was used to assess the significance of the difference, as appropriate. Measures of functional decline within one month of death were assessed using mixed effects logistic regression models.

**Results:**

The characteristics of people differed by place of death, with people who died in hospital more likely to be living at home and to not have a spouse. Less than 1 in 5 people had advance care directives or plans. Many were still being actively treated at the time of death: almost half of people who died in hospital had an investigation in their final 72 hours, less than half of people were coded as receiving palliative care at death, and more than 2 in 3 people did not get access to specialist palliative care. Declining function was associated with the terminal phase.

**Conclusion:**

This study provides novel insights for those providing end-of-life care for people with dementia. Healthcare professionals and policy makers should consider how demographic characteristics relate to the places people with dementia receive end-of-life care. The care provided to people with dementia in the last year of their life highlights the need for more support to prepare advance care documentation and timely consideration for palliative care. Changes in markers of nutritional status and function in people with advanced dementia may help with identification of terminal phases.

**Supplementary Information:**

The online version contains supplementary material available at 10.1186/s12877-023-04449-1.

## Background

Preferences for, and actual place of death are shaped by factors such as a person’s diagnosis, living situation, and level of functional independence [1]. The most common place of death for people with dementia varies between countries, and trends are changing over time [2–5]. In a population-based study in England, place of death for people with dementia who died in 2001–2010 has been found to relate to characteristics such as age, gender, marital status, and diagnoses [6]. In Australia, one in five deaths due to dementia occurs in hospital or medical service areas [7]. However, less is known about how demographic characteristics relate to place of death for Australians dying with dementia.

Where a person with dementia receives end-of-life care is important, as place of death relates to the standards and costs of the end-of-life care they receive [8]. However, across all places of death, people with dementia have been found to receive a poorer quality of end-of-life care compared to other people without dementia [9]. Sampson et al. (2006) have published the most recent retrospective case note audit study examining differences in care received by people with and without dementia who died during acute hospital admissions. The study, conducted in the United Kingdom in 2002/2003, found that people with dementia dying in hospital receive different end-of-life care to those without dementia and are less likely to be referred to palliative care, and to receive palliative medications [10]. A more recent study also found that Finnish physicians were less likely to choose a palliative care approach for a person with advanced dementia and life-threatening bleeding in 2015 than they were in 1999 [11]. Studies such as these, have indicated a need for urgent change to improve end-of-life and palliative care for people with dementia, both in Australia [12, 13], and internationally [14].

Dementia represents a group of illnesses with varied trajectories, making accurate prognostication challenging [15]. Healthcare providers and carers often have difficulty recognising when people with dementia are approaching the end of life [16], in part due to less predictable changes in function during the terminal phase of dementia, compared to other diseases [17]. People with dementia tend to gradually decline in function over time, with people often experiencing a prolonged period of low function [18]. This existing research has typically focussed on single measure tools [14, 16], and less is known about how multiple measures of functional decline might be associated with death for people in the terminal phases of dementia.

Given these developments, additional research is needed to understand the functional and clinical changes that people with dementia go through at the end of their life and investigate the care provided to people with dementia. Greater knowledge about how function declines for people with dementia in the last weeks may help to improve recognition of end of life, and in turn, help people die in their preferred place, and improve the quality of end-of-life care.

This project aimed to investigate end-of-life care for people with dementia in a regional area of Australia, specifically (1) what are the characteristics of people who die with dementia in hospital, compared to other settings; (2) what is the end-of-life care provided to people who die with dementia in hospital, compared to other settings; and (3) if there is any association between declining function and death for people with dementia.

## Methods

Clinical (or medical) audit is a valuable tool for continuous quality improvement and has been used for many years to focus on specific issues or aspects of health care and clinical practice [19]. A retrospective clinical audit was performed with the aim of comparing the care provided to people who died in public hospitals on the Central Coast, to people who died in other settings (including at home, residential aged care facilities (RACFs), and private hospitals). The Central Coast is a regional area of Greater Sydney, located on the coast of New South Wales with a population of over 348,000 [20], with more than 7,200 estimated to be living with dementia [21]. The Central Coast Local Health District provides inpatient and outpatient public health services to the local community in both hospital and community settings, and contains two acute hospitals, and two subacute hospitals. The project was deemed to be Quality Improvement and authorisation for this project was granted by the Central Coast Local Health District Research Office in May 2021 (Ref 0321-026C).

The sample included people who: 1) had a diagnosis of dementia; 2) died between 1 January 2015 and 31 December 2019, and 3) had accessed Central Coast Local Health District services prior to death. Decedents who identified as Aboriginal or Torres Strait Islander were excluded on the advice of Aboriginal stakeholders (including a representative from the Aboriginal Health and Medical Research Council), as the study did not include ethics approval from a Human Research Ethics Committee with specialist expertise in research involving Aboriginal and Torres Strait Islander Peoples.

Administrative data, including demographics, primary diagnosis, and inpatient and service events were provided by Central Coast Local Health District. Permission to obtain the data came from the District Director Quality, Strategy and Improvement who authorised the Quality Improvement application for the study on behalf of the Central Coast Local Health District, which included a waiver of informed consent. An audit tool was designed to capture additional patient characteristics, advance care documentation, consultations, cause of death, and functional decline scores, for a subset of people (see Additional file 1). We adapted the audit tool for people who died outside of hospital, as less data was available in their notes. Three pilots of the audit tool were conducted to ensure availability of data in the notes, and consistency in data extraction between researchers. A sample size calculation was performed to determine the number of records to manually audit. It was determined that approximately *n* = 300 records would provide 80% power to detect a difference in proportions of 15.9% between the group of people who died in hospital compared to people who died in other settings, with a 5% type 1 error rate.

Analysis was conducted in R version 4.2.0. Categorical descriptive variables are described with counts and frequencies with chi-square *p*-values to compare the place of death. Continuous variables are described with means and medians with 95%CI and IQR listed within the parentheses; a t-test or non-parametric test was used to assess the significance of the difference, as appropriate. Measures of functional decline within one month of death were assessed using mixed effects logistic regression models, where the outcome was defined as death within 14 days and a random intercept was included in the model to account for within person correlation. The covariates of interest in this analysis were selected based on existing literature [22, 23] and included the person's albumin scores, Ontario Modified Stratify (OMS) Fall Risk score, and Waterlow score. Albumin values were reverse coded so that the odds of death within 14 days was aligned with a declining albumin score.

## Results

A total of 705 people were identified in Central Coast Local Health District service records as dying with a dementia diagnosis between 1 January 2015 and 31 December 2019, 346 in hospital, and 359 in other settings. A subset of 299 people were selected at random. Of these, 177 people died in hospital, and 122 in other settings.

### Characteristics of people who died with dementia in hospital vs other settings

The most common primary diagnoses for people dying in the hospital compared with other settings were of the respiratory system (20% vs 12%), injury, poisoning and certain other consequences of external causes (17% vs 17%), and mental and behavioural disorders (16% vs 19%). Diseases of the respiratory system were significantly more likely to be the primary diagnosis for people dying in hospital (*P* = 0.003) (Table 1).

**Table 1:** Characteristics of people who died with dementia in hospital vs other settings in the administrative sample (*n* = 705)

Administrative sample	Hospital (*n* = 346)	Other settings (*n* = 359)	*P*-value
Age at death, mean (95%CI)	85.5 (68.0, 97.0)	84.8 (68.0, 97.0)	0.236
Female (%)	171 (49)	182 (51)	0.793
English as preferred language (%)	341 (99)	353 (98)	1.000
Born in Australia (%)	258 (75)	288 (80)	0.088
Primary diagnosis is Mental and behavioural disorders (F00-F99) (%)^b^	54 (16)	67 (19)	0.329
Primary diagnosis is Diseases of the respiratory system (J00-J99) (%)^b^	70 (20)	42 (12)	0.003^a^
Primary diagnosis is Injury, poisoning and certain other consequences of external causes (S00-T98) (%)^b^	58 (17)	62 (17)	0.937

^a^Statistically significant results ^b^Categorised using ICD-10 Clinical Modification codes. The reliability of primary diagnosis data for people who died in other settings is unknown, and findings should be interpreted cautiously

A significant difference was found in living situation, with people dying in hospital more likely to be living at home, either with family (41% vs 29%) or alone (15% vs 6%) and those dying in other settings more likely to be living in RACFs (64% vs 44%; *P* = 0.002). People without spouses listed were more likely to die in hospital (63% vs 47%), and people with spouses listed were more likely to die in other settings (53% vs 37%; *P* = 0.007) (Table 2).

**Table 2:** Characteristics of people who died with dementia in hospital vs other settings in the manual sample (*n* = 299)

Manual sample		Hospital (*n* = 177)	Other settings (*n* = 122)	*P*-value
Interpreter not required (%)		175 (99)	122 (100)	0.648
Living situation (%)	Home – with family	72 (41)	35 (29)	0.002^a^
	Home – alone	27 (15)	7 (6)	
	RACF	77 (44)	78 (64)	
	Other	1 (1)	2 (2)	
Spouse listed as a contact (%)		65 (37)	65 (53)	0.007^a^
Child listed as a contact (%)		143 (81)	96 (79)	0.765
Sibling listed as a contact (%)		14 (8)	6 (5)	0.434
Top 2 direct causes of death (%)^b^	Diseases of the respiratory system	38 (22)	-	-
	Diseases of the circulatory system	37 (21)	-	-
Top 2 antecedent causes of death (%)^b^	Diseases of the circulatory system	26 (15)	-	-
	Mental, Behavioural and Neurodevelopmental disorders	22 (12)	-	-
Top 2 other significant causes of death (%)^b^	Diseases of the circulatory system	18 (10)	-	-
	Mental, Behavioural and Neurodevelopmental disorders	10 (6)	-	-
Dementia was listed on the death certificate (%)		81 (46)	-	-

^a^Statistically significant results ^b^Categorised using ICD-10 Clinical Modification codes

Death certificates, which included cause of death, were only available for people who died in hospital. Respiratory and circulatory system diseases were most commonly listed as directly leading to death (22 and 21%). Circulatory system diseases and mental, behavioural and neurodevelopmental disorder diseases were both the most common antecedent causes of death (15% and 12%), and other significant conditions contributing to the death (10% and 6%). Dementia was only listed on 46% of the death certificates (Table 2). No significant differences were found for other variables (Tables 1 and 2).

### Advance care documentation and specialist consultations

Fewer than one in five people, both those who died in hospital and those in other settings, had advance care directives (16% vs 12%) or plans (7% vs 7%), which were created a median of 1.4 and 1.5 years and 0.9 and 0.8 years before death respectively. There were no significant differences between people who died in hospital compared with other settings (Table 3).

**Table 3:** Advance care documentation, consultations, referrals, and investigations in the manual sample (*n* = 299)

	Hospital (*n* = 177)	Other settings (*n* = 122)	*P*-value
Had an advance care directive (ACD) (%)	28 (16)	14 (12)	0.372
Years between ACD and death, Mean (95%CI)	2.1 (0.0, 7.7)	2.6 (0.1, 7.7)	0.642
Years between ACD and death, Median (IQR)	1.4 (0.2, 2.8)	1.5 (0.7, 2.6)	0.711
Had an advance care plan (ACP) (%)	12 (7)	9 (7)	1.000
Years between ACP and death, Mean (95%CI)	1.1 (0.1, 3.2)	1.6 (0.0, 5.8)	0.607
Years between ACP and death, Median (IQR)	0.9 (0.4, 1.4)	0.8 (0.3, 1.8)	0.910
Palliative care consulted in final admission (%)	37 (21)	-	-
Days between palliative consult and death, Median (IQR)	3.0 (2.0, 6.0)	-	-
Person known to Palliative Care (%)	-	39 (32)	-
Social worker involved in final admission (%)	108 (61)	-	-
Person known to Social Work (%)	-	66 (54)	-

For people who died in hospital, fewer than a third had a Palliative Care consultation during their final admission (21%), a median of 3 days before death. For deaths in other settings, almost one in three (32%) people were known to the local health district Specialist Palliative Care Service. Social workers were involved with over half of the people who died in hospital (61%), and around half of those people who died in other settings were known to Social Work (54%) (Table 3).

### End-of-life care for people who died in hospital

People were admitted under acute care types (100%), for a median time of 10 days prior to death. Most had a resuscitation plan (a medically authorised order to use or withhold resuscitation measures) completed during the final admission (96%), completed a median of 6 days before death. Few of these plans indicated the person was for rapid responses (16%). Comfort assessment charts were commenced for 68% of people, a median of 3 days before death. End of Life Pathways were commenced for 60% of people, a median of 2 days before death. Most of these documents stated that non-essential medications should be ceased (98%), and had a carer recorded (81%). Despite this, around half of people had an investigation in their final 72 hours of life (48%). When people were discharged from the service after death, their care type was most likely to be acute (52%) or palliative (44%) (Table 4).

**Table 4:** End of life plans, charts, and pathways for hospital deaths in the manual sample (*n* = 299) and administrative samples (*n* = 705)

		Hospital (*n* = 177)
Resuscitation plan completed during the final admission (%)		170 (96)
Days between resuscitation plan and death	Mean (95%CI)	16.7 (0.0, 59.5)
	Median (IQR)	6.0 (2.0, 13.0)
Resuscitation plan indicated for rapid responses (%)		28 (16)
Comfort assessment chart commenced (%)		121 (68)
Days between comfort chart commenced and death	Mean (95%CI)	5.2 (0.0, 15.3)
	Median (IQR)	3.0 (1.0, 7.0)
End of Life Pathway (EOLP) commenced (%)		106 (60)
Days between EOLP and death	Mean (95%CI)	4.8 (0.0, 22.4)
	Median (IQR)	2.0 (1.0, 5.0)
EOLP states non-essential medications ceased (%)		104 (98)
EOLP has a carer recorded (%)		86 (81)
Investigations in final 72 h (blood tests/imaging) (%)		84 (48)
		Hospital (*n* = 346)
Admitted as ‘Acute’ care type (%)		345 (100)
Length of stay	Mean (95%CI)	14.6 (1.0, 58.4)
	Median (IQR)	10.0 (5.0, 18.0)
Separation care type (%)	Rehabilitation Care	4 (1)
	Acute Care	181 (52)
	Maintenance Care	10 (3)
	Palliative Care	151 (44)

### The relationship between functional decline and death

Albumin, OMS Fall Risk, and Waterlow scores prior to death were examined for associations with death within 14 days. The logistic regression suggests there is a statistically significant association between a declining albumin score and the odds of death within 14 days. For each unit reduction in albumin score the odds of death within 14 days increased by 18.1% (OR = 1.181, 95CI: 1.084, 1.287, *p* < 0.001). For each unit increase of OMS Falls Risk Mobility the odds of death within 14 days increased by 6.2% (OR = 1.062, 95%CI: 1.032, 1.092, *p* < 0.001). For each additional Waterlow score point there was a 6.2% (OR = 1.062, 95%CI: 1.038, 1.087, *p* < 0.001) increase in the odds of death within 14 days (Table 5). The assessed performances of the models ranged from excellent discrimination (0.80 ≤ AUC < 0.90) to superior discrimination (AUC > 0.90) [24]. This result is indicative of a significant association; however the causal mechanism is outside of the scope of this analysis.

**Table 5:** Logistic regression model Albumin, OMS Falls Risk Mobility, and Waterlow scores in the manual sample (*n* = 299)

	Number of people	Number of Measurements	Est	OR	LCL	UCL	Std. Error	Z value	Pr( >|z|)	AUC
Albumin^a^	200	654	0.17	1.181	1.084	1.287	0.04	3.81	< 0.001	0.958
OMS Falls Risk Mobility^b^	189	976	0.06	1.062	1.032	1.092	0.01	4.12	< 0.001	0.898
Waterlow^c^	166	1906	0.06	1.062	1.038	1.087	0.01	5.18	< 0.001	0.881

OR are calculated from separate crude logistic regression models for functional decline measurements within 1 month of death

^a^albumin blood test (used to assess general health and liver and kidney function)

^b^Ontario Modified Stratify (OMS) (Sydney Scoring) Falls Risk Mobility Score (identifies people’ falls risk factors)

^c^Waterlow Score (an assessment of a person’s risk of developing a pressure injury)

## Discussion

This retrospective case note audit of people who died with dementia explored the end-of-life care they received. The findings provide novel insights, including, the relationship between place of death and demographic characteristics, the care provided, and the association between functional decline and death.

People dying in hospital were found to be more likely to be living at home (either with family or alone), and to not have a spouse listed as a contact. Those dying in settings outside the hospital were more likely to be living in RACFs, and to have a spouse listed as a contact. Other studies have also shown that place of death with dementia relates to marital status [9]. These findings support the notion that spousal carer support is needed to achieve a death outside of hospital, and that additional support may be needed for those without a spouse who have a preference to die outside the hospital. A previous study reported that comorbidities correlate with an increased probability of people with dementia dying in hospital [25]. We add new evidence that respiratory system diseases were significantly more likely to be the primary diagnosis for people dying in hospital compared to other settings. The finding that people dying outside of hospital were more likely living in RACFs highlights the importance of residential care facilities as a place of end-of-life care, and the need to adequately educate and resource these facilities to allow residents with dementia to die well.

Interestingly, only around half of people who died with dementia had this diagnosis listed on their death certificate, which is similar to other population studies in Australia [26]. Significant underreporting of dementia on death certificates has also been identified internationally [27]. This may reflect that dementia may not be the direct cause of death and people with dementia die due to other comorbidities. On the other hand, it may reflect the misunderstanding, myths or denial in that people and healthcare professionals do not accept dementia as a terminal disease, although some research suggests that awareness around this may be increasing [28]. It is beyond the scope of this study to determine the reasons for absence of dementia in death certification and it certainly warrants further investigation and discussion.

Comfort assessment charts were commenced for 68% of people, a median of 3 days before death, and End of Life Pathways were commenced for 60% of people, a median of 2 days before death. These findings suggest that the terminal phase was recognised reasonably late. Not surprisingly, only one in five people who died in hospital had a specialist palliative care referral during that final admission, a median of 3 days before death. This implies that access to specialist palliative care is limited and delayed. Others have suggested that these issues with specialist palliative care access are the result of a lack of consensus regarding referral criteria for people with dementia and argue for standardised referral processes [29]. These barriers to palliative care are reflected in the finding that almost half of people had an investigation in their final 72 h of life, indicating that impending death had either not been recognised by the treating team or that there was uncertainty regarding level of treatment desired.

Poor rates of advance care planning and resulting burdensome interventions have been longstanding issues in dementia care [30]. Our results demonstrate, with fewer than one in five people dying in hospital and other settings having advance care directives or plans, this issue remains contemporary. Lack of advance care planning is a well-established barrier to the provision of palliative care for people with advanced dementia [2]. Advance care planning discussions shortly after a dementia diagnosis have been evaluated positively by people and carers [31]. Strategies are needed to ensure these discussions are part of routine clinical practice.

Social workers were involved in the end-of-life care for around half of people with dementia. A previous study has found social workers play a critical role in providing end-of-life care to people with dementia [32]. Further research would help understand the role of social workers in multidisciplinary care teams providing palliative and end-of-life care to people with dementia [33].

Previous research has explored how functional decline might help us to identify end of life in people with dementia. For example, Sampson et al.’s 2013 prospective cohort study which found the effect of dementia on mortality was reduced after adjustment for Waterlow score [34]. Similarly, Morgan et al.’s 2019 Australian consecutive cohort study using the Australia-modified Karnofsky Performance Status Scale, found the most rapid rate of decline occurs in the last 2 weeks of life for all cohorts, including the dementia cohort [35]. This study reports additional evidence that significant associations were found between changes in albumin, OMS Falls Risk Mobility, and Waterlow scores and death within 14 days, and these models had excellent discrimination. These findings suggest that changes in markers of nutritional status and function in people with dementia may help healthcare providers to identify terminal phases.

This study has limitations. Firstly, the data did not distinguish between home and RACF deaths as these were combined as non-hospital deaths. The Central Coast Local Health District where the data were collected, has distinctive demographic characteristics, such as a population with a higher median age, lower level of educational attainment, and higher percentage of people born in Australia compared to the New South Wales state and Australia [36]. This may limit the generalisability of the results. Secondly, decedents who identified as Aboriginal or Torres Strait Islander were not included in the data. Given high growth in the number of Aboriginal and Torres Strait Islander people living with dementia, urgent investment is needed in the development of culturally appropriate end-of-life dementia care [37]. Thirdly, while the markers explored here had high discrimination (as reflected in high AUCs), this does not mean that the models are necessarily well calibrated. Models can discriminate well between those who are at higher risk than others, but not assign an absolute risk of mortality accurately. Future research should investigate calibration and whether the absolute risk predicted matches the observed risk. Larger samples are needed to explore which thresholds maximise sensitivity or specificity.

## Conclusion

This study reported on the palliative and end-of-life care provided to people with dementia, including consideration of how social demographic characteristics shape the location of end-of-life care. The findings highlight that early preparation of advance care documentation and clarity around referrals to specialist palliative care, remain areas for improvement in advanced dementia care. Importantly, the study provides insight into how identification of functional decline may help clinicians identify when the last weeks of life are approaching. To improve end of life for people with dementia, future studies should develop and test dementia-specific models of care for end of life [38], which are underpinned by in-depth understanding of clinical, functional, and social changes occurring during the last weeks and months of life.

### Supplementary Information

**Additional file 1.** Audit Tool.

## Data Availability

The data that support the findings of this study are available from Central Coast Local Health District, but restrictions apply to the availability of these data, which were used under authorisation for the current study, and so are not publicly available. Data are however available from the authors upon reasonable request and with permission of Central Coast Local Health District.

## Abbreviations

RACFs: Residential aged care facilities
OMS: Ontario Modified Stratify
