# Supplementary material for: What care do people with dementia receive at the end of life? Lessons from a retrospective clinical audit of deaths in hospital and other settings
Source: BMC Geriatr. 2024 Jan 9;24:40. doi: 10.1186/s12877-023-04449-1 (PMC10775581; doi:10.1186/s12877-023-04449-1)
Supplement: Supplementary file 1 — Additional file 1. Audit Tool. [file 12877_2023_4449_MOESM1_ESM.docx]

**Additional File 1: Audit Tool**

For patients who died in a CCLHD facility:

- Living situation
- Interpreter required
- Referrals for BPSD
- Patient contacts listed
- Was a resuscitation plan completed during the final admission?
  - If YES, Date of final resuscitation plan
  - If YES, Did the final resuscitation plan indicate they were still for rapid responses?
- Was there an advanced care directive?
  - If YES, ACD date
- Was there an advanced care plan?
  - If YES, ACP date
- Comfort assessment chart commenced?
  - IF YES, Date Comfort assessment chart commenced?
- Was an End of Life Care Pathway commenced?
  - If YES, Date EOLCP commenced
  - Non-essential medications ceased?
  - Was a carer recorded in the EOLCP?
- Was the palliative care team consulted in the final admission?
  - IF YES, Palliative care consult date
- Social worker involved in the final admission
- Cause of death
  - If dementia is listed on the death certificate, how long with disease?
- Were there investigations in final 72 hours? (Blood tests / imaging)
- Waterlow scores and dates
- Albumin scores and dates
- OMS Falls Risk scores and dates

For patients who died outside of a CCLHD facility:

- Living situation
- Interpreter required
- Referrals for BPSD
- Patient contacts listed
- Was there an advanced care directive?
  - If YES, ACD date
- Was there an advanced care plan?
  - If YES, ACP date
- Was the patient known to the palliative care team?
- Was the patient known to social work?
- Waterlow scores and dates
- Albumin scores and dates
- OMS Falls Risk scores and dates
